# Drug*placebo interaction effect may bias clinical trials interpretation: hybrid balanced placebo and randomized placebo-controlled design

**DOI:** 10.1186/s12874-016-0269-1

**Published:** 2016-11-29

**Authors:** Muhammad M. Hammami, Safa Hammami, Reem Al-Swayeh, Eman Al-Gaai, Faduma Abdi Farah, Sophia J. S. De Padua

**Affiliations:** 1 Department of Clinical Studies and Empirical Ethics, King Faisal Specialist Hospital and Research Center, P O Box # 3354 (MBC 03), Riyadh, 11211 Saudi Arabia; 2 Alfaisal University College of Medicine, Riyadh, Saudi Arabia

**Keywords:** Balanced placebo design, Randomized placebo-controlled trials, Biased drug effect size, Drug placebo interaction, Effect modification

## Abstract

**Background:**

Conventional randomized placebo-controlled study design assumes the absence of drug*placebo interaction. We hypothesized the presence of such an interaction and that conventionally estimated drug effect might be biased. The objectives of the study were to determine the drug*placebo interaction effect (main) and compare conventionally estimated and interaction model-estimated drug effects (secondary).

**Methods:**

We used a hybrid of balanced placebo and randomized placebo-controlled designs. Four hundred eighty healthy volunteers were randomized to three groups. The first received hydroxyzine (25 mg) described as hydroxyzine or placebo, the second received placebo described as hydroxyzine or placebo, and the third received hydroxyzine and placebo described as unknown; each in a randomized crossover design. Seven participants failed to crossover. Group assignment was concealed from participants and study coordinators. Coordinators were blinded to group and intervention assignment. Participants and coordinators were deceived as to study objectives. Main outcomes were mean area-under-the-curve of drowsiness (therapeutic outcome) and mouth-dryness (adverse outcome), self-reported on 100 mm visual analog scale over 7 h. Drug, placebo, placebo + interaction, and total effects were estimated using analysis of covariance by comparing received hydroxyzine/told placebo to received placebo/told placebo, received placebo/told hydroxyzine to received placebo/told placebo, received hydroxyzine/told hydroxyzine to received hydroxyzine/told placebo, and received hydroxyzine/told hydroxyzine to received placebo/told placebo, respectively. Drug effect was also conventionally estimated in the third group.

**Results:**

Mean (SD) age was 31.4 (6.6) years, 65% were males. There was significant difference between placebo + interaction effect and placebo effect for both drowsiness and mouth-dryness with a mean difference (95% confidence interval) of 35.1 (5.6 to 64.6) and 23.8 (2.4 to 45.2) mm*hr, respectively. Total effect was larger than the sum of drug and placebo effects for drowsiness (139.7 (109.8 to 169.6) vs. 99.1 (68.2 to 130.0) mm*hr) and mouth-dryness (63.6 (41.1 to 86.1) vs. 34.7 (11.1 to 58.4) mm*hr). Conventionally estimated drug effect was larger than interaction model-estimated drug effect for drowsiness (69.2 (45.5 to 92.8) vs. (58.3 (31.6 to 85.0) mm*hr) and mouth-dryness (19.9 (5.3 to 34.5) vs. 9.5 (−9.2 to 28.1) mm*hr).

**Conclusions:**

There is significant and important drug*placebo interaction effect that may bias conventionally estimated drug effect.

**Trial registration:**

ClinicalTrial.gov identifier: NCT01501591 (registered December 25, 2011).

## Background

The drug effect of a medication is conventionally best estimated by comparing the placebo and medication arms of randomized placebo-controlled trials (RPCT). The comparison can be made on an additive scale or multiplicative scale, i.e., calculating the difference or ratio of the results in the two arms, respectively.

Changes observed in the placebo arm, referred to as “placebo” response, results from the meaning response (placebo effect) [1] and non-specific passive changes [2, 3], such as regression to the mean and Hawthorne effect.

Interventions are described as “additive” if their effects do not interact and as “non-additive” if their effects reciprocally modify each other. The interaction or effect modification could be positive or negative and could be seen on one or both of the additive and multiplicative scales.

Participants in the medication arm of an RPCT are exposed to both the drug effect and the placebo effect, whereas participants in the placebo arm are exposed to the placebo effect only. Regardless of whether the additive or multiplicative scale is used, the common interpretation of RPCTs is based on the assumption that the drug effect and the placebo effect are “additive”, i.e., there is no drug*placebo interaction or effect modification [3]. Thus to estimate the drug effect, changes observed in the placebo arm are simply “subtracted” from those observed in the medication arm. If a drug*placebo interaction effect does exist, it would be restricted to the medication arm, the difference between the two arms would be more or less than the drug effect, and conventionally estimated drug effect would be biased [4, 5].

Balanced placebo design, where subjects receiving a medication or placebo are either told that they are receiving the medication or placebo [6], can model total medication effect into drug, placebo, and drug*placebo interaction effects [7]. However, because of the (mis) information provided to participants, the design raises substantial ethical concerns and may raise suspicion among participants [8]. We have previously used a modification of the balanced placebo design, namely, balanced placebo crossover design to study the drug*placebo interaction effect [9]. The crossover version and other novel designs were later advocated to overcome the shortcomings of the balanced placebo design [8]. Nevertheless, even the balanced placebo crossover design has ethical constraints. Further, it does not allow direct comparison of interaction model-estimated drug effect and conventionally estimated drug effect.

In the current study, we used a hybrid of balanced placebo crossover design and RPCT design to estimate the drug*placebo interaction effect (primary aim) and compare interaction model-estimated and conventionally estimated drug effects (secondary aim). We recruited healthy volunteers and used hydroxyzine, a medication with known safety profile, to minimize ethical concerns. Primary outcomes were a therapeutic effect (drowsiness) and an adverse effect (mouth-dryness) of hydroxyzine. To verify that study procedures are effective in inducing and measuring the placebo effect, our study design included two control outcomes, nausea as a positive control (participants were told that hydroxyzine causes nausea, but it doesn’t), and itchiness as a negative control (participants were told that hydroxyzine doesn’t cause itchiness, and it doesn’t).

## Methods

### Design

**Fig. 1:**
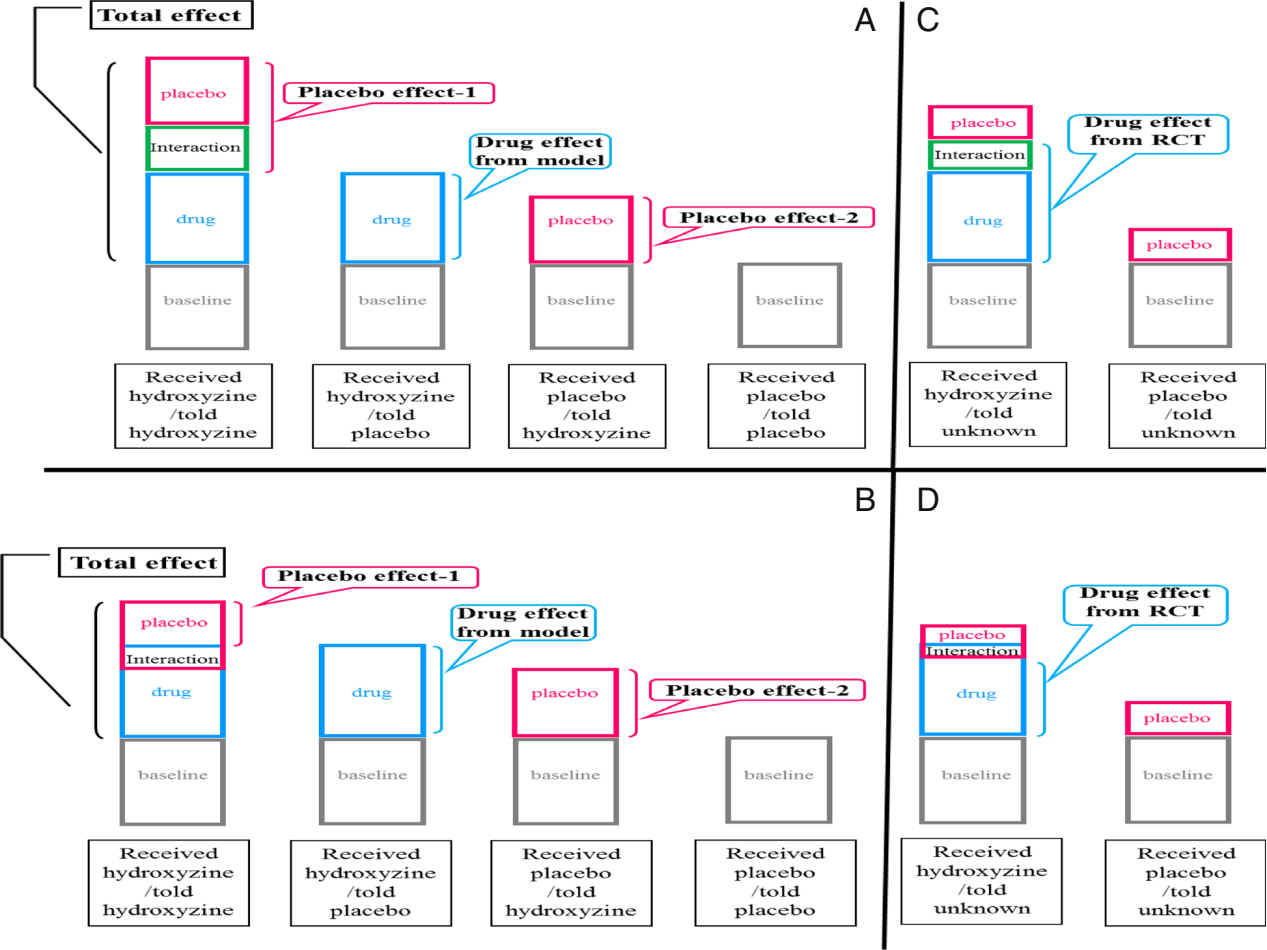
Models of Positive and Negative Drug*Placebo Interaction Effect in Balanced Placebo and Conventional Randomized Placebo-Controlled Designs. **a** and **b** depict balanced placebo design. **c** and **d** depict conventional randomized placebo-controlled design. A positive drug*placebo interaction model is presented in **a** and **c**. A negative drug*placebo interaction model is presented in **b** and **d**. Hydroxyzine is used as the model drug. **a & b** Under the balanced placebo design, ooutcome measures in received placebo/told placebo represent none-specific passive changes (baseline); in received placebo/told hydroxyzine, placebo effect and baseline; in received hydroxyzine/told placebo, drug effect and baseline; and in received hydroxyzine/told hydroxyzine, drug, placebo, drug*placebo interaction effects, and baseline. The total effect of ingesting a hydroxyzine capsule is the difference between received hydroxyzine/told hydroxyzine and received placebo/told placebo and consists of drug, placebo, and drug*placebo interaction effects. The interaction model-estimated drug effect is the difference between received hydroxyzine/told placebo and received placebo/told placebo and consists of the drug effect only. The model predicts two different estimates of the placebo effect: placebo effect-1, the difference between received hydroxyzine/told hydroxyzine and received hydroxyzine/told placebo, which consists of the placebo and the drug*placebo interaction effects; and placebo effect-2, the difference between received placebo/told hydroxyzine and received placebo/told placebo, which consists of the placebo effect only. Placebo effect-1 is larger than placebo effect-2 if there is positive interaction effect (**a**) and smaller if there is negative interaction effect (**b**). **c & d** Under the conventional randomized placebo-controlled design, outcome measures in received hydroxyzine/told unknown represent the drug effect, part of the placebo effect (because of participants’ knowledge that there is 50% chance of receiving hydroxyzine) and consequently part of the drug*placebo interaction effect, and baseline; and in received placebo/told unknown, part of the placebo effect (because of participants’ knowledge that there is 50% chance of hydroxyzine) and baseline. Conventionally estimated drug effect is the difference between received hydroxyzine/told unknown and received placebo/told unknown and consists of the drug effect and part of the interaction effect. It is larger than the interaction model-estimated drug effect if there is positive interaction effect (compare **a** and **c**) and smaller if there is negative interaction effect (compare **b** and **d**)

The design was a hybrid of crossover-balanced placebo and RPCT designs. In effect, participants were randomized to three groups or three randomized crossover arms. The first group received hydroxyzine twice, described as hydroxyzine or placebo, the second group received placebo twice, described as hydroxyzine or placebo, and the third group received hydroxyzine and placebo described as unknown (Fig. 1).

### Participants

Healthy volunteers were recruited via advertisement throughout the King Faisal Specialist Hospital and Research Center (KFSH&RC). Main eligibility criteria were an age of 18–50 years (based on safety reasons) and ability to use visual analog scales (VAS) reproducibly. The study was conducted at KFSH&RC from November 2012 through October 2015 between 8:00–8:30 am to 3:00–3:30 pm. It followed ethical guidelines on deception use in research [10, 11] and was approved by KFSH&RC Research Ethics Committee. Participants gave a written “consent”, being told that the study compares 25 mg hydroxyzine to placebo to determine how much of the hydroxyzine-related symptoms aren’t caused by hydroxyzine, and that they have a 50/50 chance of receiving hydroxyzine or placebo. They were compensated based on the Wage-Payment model [12] and were contacted post-study for debriefing on the actual study purpose and design and for delayed full consenting. Headache was reported by seven participants (three received hydroxyzine, four received placebo), back pain by one, fever by two, and left-sided chest warmness by one (all received hydroxyzine). All symptoms were mild and resolved spontaneously.

### Procedures and interventions

Participants were reminded to abstain from smoking, caffeine, and alcohol for 16 h, from food for 10 h, and from water for 1 h, and to have ≥7 h of good sleep before each study period. On study day, baseline data were obtained and a standardized breakfast was served, followed 15–30 min later by intervention administration. Participants remained ambulatory or seated upright for 4 h, were allowed to drink ≤120 ml of water hourly, and were served standardized lunch at 4 h.

Intervention administration (by MMH) involved individually informing participants that hydroxyzine (but not placebo) may cause drowsiness, mouth-dryness, and nausea, but not itchiness; asking them to carefully read and sign another “consent” statement that included study purpose, expected symptoms, and current assignment; and watching them swallow one hydroxyzine or placebo capsule dispensed from containers with clear, purposely-displayed labels. Group-1 received two 25 mg hydroxyzine capsules (described as hydroxyzine or placebo) 72 h apart, group-2, received two placebo capsules (described as hydroxyzine or placebo) 72 h apart, and group-3, one hydroxyzine capsule and one placebo capsule (described as unknown with 50/50 chance of being hydroxyzine or placebo) 72 h apart.

Hydroxyzine, clinically used for sedation and treatment of pruritus and nausea, was chosen for its safety profile and subjective effects. Sedation usually starts within 1 h and persists for 6 h, plasma half-life is around 20 h, and mouth-dryness is rather common. [13] A 25 mg dose was selected to avoid a ceiling effect that would reduce the ability to detect a positive drug*placebo interaction effect. Hydroxyzine capsules were manufactured from commercially available 25 mg hydroxyzine hydrochloride tablets and contained similar amount of lactose as the color- and shape-matched placebo capsules. Hydroxyzine concentration was blindly measured by high performance liquid chromatography assay [14].

### Outcome measures

Co-primary outcome measures were area-under-the-curve (AUC) of drowsiness and mouth-dryness levels assessed on 100 mm horizontal VAS at baseline and 0.5, 1.0, 1.5, 2.0, 2.5, 3.0, 4.0, 5.0, 6.0, and 7.0 h after administering the intervention. Levels of nausea and itchiness were assessed in the same way to be used as positive and negative control outcomes, respectively, of the placebo effect. As such, we did not consider them primary outcomes to be registered at clinicaltrial.gov. However, they were stated in the original proposal. Each VAS was anchored by word descriptors at each end, and scores were determined in mm. Secondary outcome measures were mean number of times drowsiness and mouth-dryness were reported on a binary scale at the same time points. At each time point, data collection sheets were handed to participants and then collected and checked for completeness by coordinators.

### Randomization

Randomization schedule was generated (by MMH) using a program available on-line (www.randomization.com). We block-randomized (block size = 6) 480 slots to six assignments: hydroxyzine described as hydroxyzine followed by hydroxyzine described as placebo, hydroxyzine described as placebo followed by hydroxyzine described as hydroxyzine (group-1), placebo described as placebo followed by placebo described as hydroxyzine, placebo described as hydroxyzine followed by placebo described as placebo (group-2), blinded hydroxyzine followed by blinded placebo, and blinded placebo followed by blinded hydroxyzine (group-3). Group assignment was concealed from participants and study coordinators.

### Blinding and deception

Study coordinators were blinded to group and intervention assignments and were deceived as to study objectives. Participants were deceived as to study objectives and partially deceived (group-1 and group-2) or blinded (group-3) to individual assignment. To enhance blinding/deception and verify compliance, blood samples for hydroxyzine concentration were drawn at 3 h, and participants were requested not to reveal their assignments to the coordinators. To assess blinding, the coordinators were asked to guess the intervention (hydroxyzine or placebo) at 1 h. To assess deception, participants were asked during post-study debriefing whether they believed their intervention assignment or guessed actual study objectives and design.

### Interaction model

The interaction model assumes that outcome measures in participants who receive placebo and are told that it is inert (received placebo/told placebo), represent non-specific passive changes. These measures are used as reference (baseline) since they do not include a drug effect or a placebo effect. In participants who receive placebo but are told that it is an active drug (received placebo/told drug), there would be a placebo effect but no drug effect. In participants who receive an active drug and are told that it is active drug (received drug/told drug), there would be a drug effect, placebo effect, and drug*placebo interaction effect. In participants who receive an active drug but are told that it is inert (received drug/told placebo), there would be a drug effect but no placebo effect; the placebo effect would be “subtracted.” In RPCT, participants typically know that they have 50% chance of receiving an active drug and 50% chance of receiving a placebo. If they do receive an active drug (received drug/told unknown), there would be a full drug effect, part of the placebo effect, and consequently, part of the drug*placebo interaction effect. If they do receive a placebo (received placebo/told unknown), there would be part of the placebo effect and no drug effect. Based on the model, the following estimations can be made:

1. Total effect is the difference between received drug/told drug and received placebo/told placebo; it consists of the drug effect, placebo effect, and drug*placebo interaction effect.
2. Interaction model-estimated drug effect is the difference between received drug/told placebo and received placebo/told placebo; it consists of the drug effect only.
3. Drug + interaction effect is the difference between received drug/told drug and received placebo/told drug; it consists of the interaction model-estimated drug effect and the drug*placebo interaction effect.
4. Conventionally estimated drug effect is the difference between received drug/told unknown and received placebo/told unknown; it consists of the drug effect and part of the interaction effect.
5. Placebo effect plus interaction effect (here called placebo effect-1) is the difference between received drug/told drug and received drug/told placebo; it consists of the placebo effect and the drug*placebo interaction effect.
6. Placebo effect only (here called placebo effect-2) is the difference between received placebo/told drug and received placebo/told placebo; it consists of the placebo effect only.

The model predicts that if there is a positive drug*placebo interaction effect, placebo effect-1 would be larger than placebo effect-2, total effect would be larger than the sum of the interaction model-estimated drug effect and placebo effect-2, and conventionally estimated drug effect would be larger than interaction model-estimated drug effect. The opposites would be expected with negative interaction. Thus, the model appropriately addresses the primary and secondary aims of the study, i.e., whether placebo effect-1 is different from placebo effect-2 and whether interaction model-estimated drug effect is different from conventionally estimated drug effect. The model also predicts that there would be drug, placebo, and drug*placebo interaction effects on drowsiness and mouth-dryness, only placebo effect on nausea, and no drug or placebo effects on itchiness. The positive and negative interaction models are presented in Fig. 1 using the study drug, hydroxyzine, as example.

### Sample size

The study was designed to have adequate power to compare placebo + interaction (placebo-1) and placebo only (placebo-2) effects in group-1 and group-2, respectively (primary aim). It was planned to recruit 160 subjects in each group, based on an expected standardized mean difference of 0.33 [9], type I error of 0.05, type II error of 0.2, and 10% dropout rate. An additional 160 subjects (group-3) were included to measure conventionally estimated drug effect (secondary aim).

### Statistical analysis

Outcome measures were evaluated by analysis of covariance. For within-group analysis (to estimate placebo effect-1, placebo effect-2, and conventionally estimated drug effects), statistical model included intercept, baseline value, period, intervention, group, and subject-nested within group. The last two terms were omitted for between-group analysis (to estimate drug + interaction, total, and interaction model-estimated drug effects). Comparison of estimated effect sizes was performed by the z test; the combined standard error was calculated as the square root of sum of squares of the separate standard errors [15]. Predefined primary and secondary outcomes were differences between placebo effect-1 and placebo effect -2 and between conventionally estimated and model-estimated drug effects, respectively. Non-parametric tests were used for secondary analysis. Analyses were performed (by MMH) with IBM SPSS Statistics version 21 software. Two-tailed p-values and 95% confidence intervals are reported. Analyses were not adjusted for multiple comparisons.

## Results

**Fig. 2:**
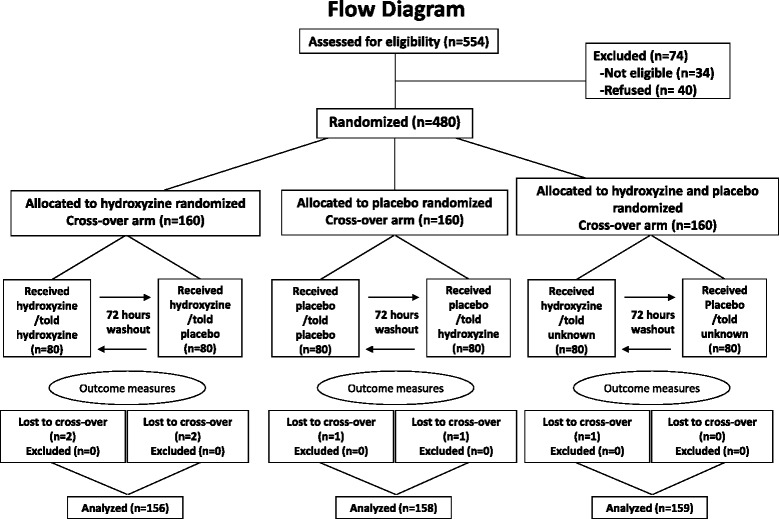
Flow of Participants through the Study

We screened 554 volunteers and found 34 ineligible (13 due to medical history, 11 to laboratory results, 10 to failing the VAS screening test). Forty of the 520 eligible volunteers did not show up; 480 were randomized. Seven participants lost to crossover for personal reasons and were excluded from analysis (Fig. 2). Study coordinators correctly guessed participants’ assignment (hydroxyzine vs. placebo) 52% of the time. We were able to contact 266 (56%) out of the 473 participants for post-study debriefing, all gave full informed consent. Out of the 266, 264 (99%) were unable to guess the actual study objectives or design, and 129 were assigned to group-1 or group-2. Out of the 129, 118 (91%) believed what they were told at the time of administering the intervention (two did not, nine had doubt), and 102 (80%) believed it at all times (13 did not, 14 had doubt), indicating successfulness of blinding and deception. Plasma hydroxyzine concentrations confirmed compliance of all participants.

**Fig. 3:**
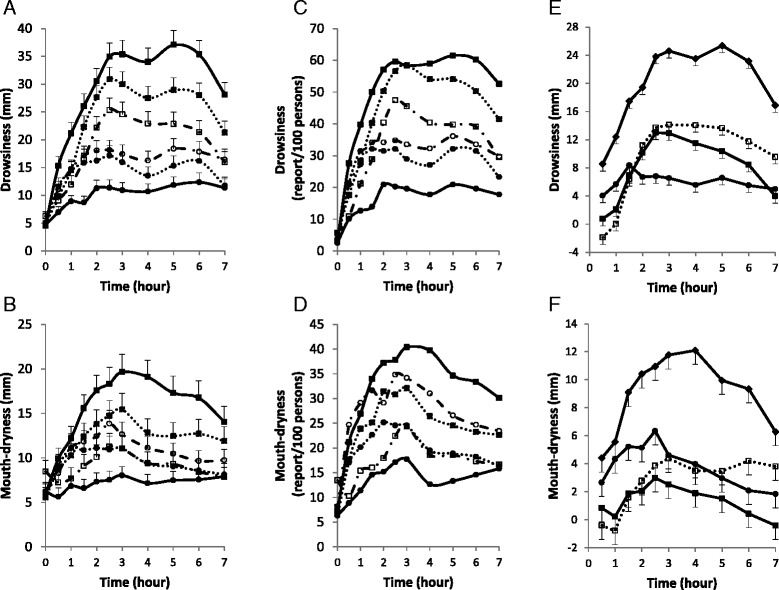
Time Course of Self-Reported Drowsiness and Mouth-dryness According to Intervention or Type of Effect. **a** to **d** mean unadjusted scores on continuous scale (**a** and **b**) and binary scale (**c** and **d**) per intervention. *Squares* indicate receiving 25 mg hydroxyzine, described as hydroxyzine (*closed square with continuous line*), as placebo (*open square with interrupted line*), or as unknown (*closed square with dotted line*). *Circles* indicate receiving placebo, described as placebo (*closed circle with continuous line*), as hydroxyzine (*open circle with interrupted line*), or as unknown (*closed circle with dotted line*). **e** and **f** mean adjusted scores per type of effect. *Closed diamonds* indicate total effect; *closed squares* interaction model-estimated drug effect, *open squares* conventionally estimated drug effect, and *closed circle* placebo effect. *T-bars* indicate standard errors

Overall mean (SD) age was 31.4 (6.6) years, and 309/473 (65%) were males. Table 1 summarizes participants’ characteristics per assignment. Baseline differences of outcome measures among the six assignments were not significant (*p* = 0.10–0.74). Figure 3 depicts unadjusted mean (SE) VAS scores of drowsiness and mouth-dryness (A and B) and unadjusted mean number of times the two symptoms were reported (C and D).

**Table 1:** Baseline characteristics of study participants

Characteristics	Hydroxyzine Group		Placebo Group		Blinded Hydroxyzine/Placebo Group	
Age, mean (SD), yr.	31.5 (6.4)		30.8 (6.1)		31.8 (7.2)	
Sex, no. (%)						
Female	50 (32)		56 (35)		58 (36)	
Male	106 (68)		102 (65)		101 (64)	
Completed education, no. (%)						
High school	36 (23)		38 (24)		30 (19)	
College	85 (55)		72 (46)		88 (55)	
University	35 (22)		48 (30)		41 (26)	
Occupational status, no. (%)						
Professional, technical, managerial	32 (22)		45 (29)		37 (24)	
Clerical, sales	1 (1)		0 (0)		2 (1)	
Service	107 (72)		99 (63)		101 (66)	
Agricultural, fishery, related	0 (0)		1 (1)		2 (1)	
House wife	0 (0)		1 (1)		2 (1)	
Student	5 (3)		6 (4)		7 (5)	
Unemployed	4 (3)		4 (3)		3 (2)	
	Told hydroxyzine	Told placebo	Told placebo	Told hydroxyzine	Received hydroxyzine	Received placebo
Drowsiness level, mean (SD), mm^a^	4.5 (6.0)	6.3 (10.1)	4.7 (6.6)	5.1 (8.5)	4.9 (7.3)	4.6 (8.0)
Mouth-dryness level, mean (SD), mm ^a^	5.9 (9.5)	8.5 (15.5)	6.2 (10.2)	6.1 (11.5)	5.5 (8.8)	5.7 (11.6)
Nausea level, mean (SD), mm ^a^	2.8 (4.4)	3.0 (5.8)	2.9 (4.9)	3.5 (7.4)	2.3 (5.2)	3.0 (6.4)
Itchiness level, mean (SD), mm ^a^	2.8 (3.8)	2.6 (4.3)	2.8 (4.5)	3.1 (5.3)	2.7 (4.5)	2.7 (4.4)

Hydroxyzine group (group-1, *n* = 156): received 25 mg hydroxyzine twice and told its hydroxyzine on one time and placebo on the other, in a randomized crossover design. Placebo group (group-2, *n* = 158): received placebo twice and told its placebo on one time and 25 mg hydroxyzine on the other, in a randomized crossover design. Blinded hydroxyzine/placebo group (group-3, *n* = 159): received 25 mg hydroxyzine and placebo in a randomized single-blinded crossover design. Numbers may not sum up to group totals because of missing data and percentages may not add to 100% because of rounding

^a^ Measured on 100 mm visual analog scale

Differences between period 1 and period 2 were significant for baseline drowsiness (mean difference 1.5 mm, *p* < 0.001), mouth-dryness (4.4 mm, *p* < 0.001), nausea (0.8 mm, *p* = 0.002), but not itchiness (0.3 mm, *p* = 0.18). Further, in analysis of covariance, there was significant period effect on mean AUC of drowsiness (25.3 mm*hr, *p* < 0.001), mouth-dryness (16.2 mm*hr, *p* < 0.001), nausea (6.7 mm*hr, *p* = 0.003), and itchiness (3.4 mm*hr, *p* = 0.02); and on mean number of times drowsiness (*p* = 0.01), mouth-dryness (*p* = 0.003), nausea (*p* = 0.006), and itchiness (*p* = 0.007) were reported. Therefore, all estimates were adjusted for the period effect.

There was also significant overall intervention effect on mean AUC of drowsiness, mouth-dryness, and nausea (*p* < 0.001) but not itchiness (*p* = 0.93) and on mean number of times drowsiness, mouth-dryness, and nausea were reported (*p* < 0.001).

### Drug effect

**Fig. 4:**
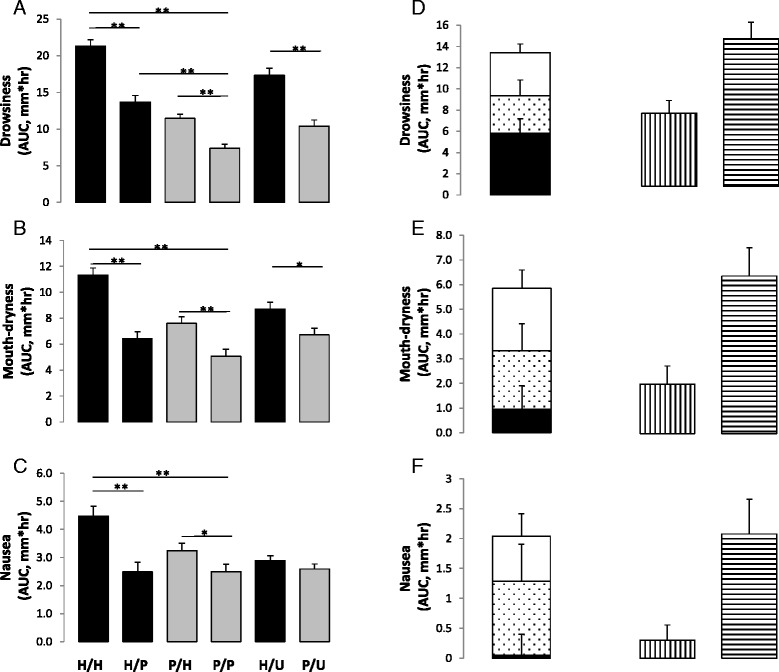
Mean Area-Under-the-Curve According to Intervention or Type of Effect. **a** to **c** adjusted mean area-under-the-curve after receiving 25 mg hydroxyzine (*black bars*), described as hydroxyzine (H/H), as placebo (H/P), or as unknown (H/U); or placebo (*grey bars*), described as hydroxyzine (P/H), as placebo (P/P), or as unknown (P/U). **d** to **f** adjusted interaction model-estimated drug effect (*black bars*), drug*placebo interaction effect (*dotted bars*), placebo effect (*white bars*), conventionally estimated drug effect (*vertical strips bars*), and total effect (*horizontal strips bars*). *T-bars* indicate standard errors. * *p* <0.05, ** *p* <0.001

Adjusted mean (95% confidence interval) AUC of model-estimated and conventionally-estimated drug effects were, respectively, 58.3 mm*hr (31.6 to 85.0, *p* < 0.001) and 69.2 mm*hr (45.5 to 92.8, *p* < 0.001) on drowsiness, 9.5 mm*hr (−9.2 to 28.1, *p* = 0.32) and 19.9 mm*hr (5.3 to 34.5, *p* = 0.008) on mouth-dryness, 0.5 mm*hr (−6.4 to 7.4, *p* = 0.89) and 3.0 mm*hr (−1.9 to 8.0, *p* = 0.23), on nausea (Fig. 4 (a to c)), and 2.5 mm*hr (−6.0 to 11.0, *p* = 0.56) and -0.7 mm*hr (−4.5 to 3.2, *p* = 0.73) on itchiness. The results indicate that the RPCT overestimates drug effect by about 19, 109, and 500% for drowsiness, mouth-dryness, and nausea, respectively (Fig. 3 (e & f) and Fig. 4 (d to f)). Interestingly, outcome measures in received hydroxyzine/told unknown were intermediate between those in received hydroxyzine/told hydroxyzine and those in received hydroxyzine/told placebo (Figs. 3 and 4).

Using non-parametric tests, unadjusted conventionally estimated drug effect was significant for AUC of drowsiness (*p* < 0.001), mouth-dryness (*p* = 0.01), but not nausea (*p* = 0.60), whereas, model-estimated drug effect was significant for AUC of drowsiness (*p* < 0.001) but not mouth-dryness (*p* = 0.21) or nausea (*p* = 0.73).

### Placebo effect

Adjusted mean AUC of placebo effect-1 and placebo effect-2 were, respectively, 75.9 mm*hr (50.8 to 101.0, *p* < 0.001) and 40.8 mm*hr (24.9 to 56.7, *p* < 0.001) for drowsiness, 49.1 mm*hr (33.3 to 64.8, *p* < 0.001) and 25.3 mm*hr (10.5 to 40.0, *p* = 0.001) for mouth-dryness, 19.9 mm*hr (10.1 to 29.7, *p* < 0.001) and 7.5 mm*hr (0.1 to 14.9, *p* = 0.047) for nausea (Fig. 4 (a to c)), and 0.5 mm*hr (−6.7 to 7.7, *p* = 0.89) and 1.6 mm*hr (−2.0 to 5.3), *p* = 0.39) for itchiness. Further, adjusted placebo effect-1 and placebo effect-2 on binary scale (mean number of reports per 100 persons) were, respectively, 182 (121 to 243, *p* < 0.001) and 143 (89 to 197, *p* < 0.001) for drowsiness, 158 (103 to 213, *p* < 0.001) and 147 (86 to 209, *p* < 0.001) for mouth-dryness, and 76 (36 to 116, *p* < 0.001) and 36 (7 to 65, *p* = 0.01) for nausea.

Using non-parametric tests, placebo effect-1 was significant for AUC of drowsiness (*p* < 0.001), mouth-dryness (*p* < 0.001), and nausea (*p* < 0.001), whereas, placebo effect-2 was significant for AUC of drowsiness (*p* < 0.001), mouth-dryness (*p* < 0.001), but not nausea (*p* = 0.08). There was significant correlation of placebo effect-1 on mouth-dryness and drowsiness (rho = 0.52, *p* < 0.001) and on mouth-dryness and nausea (rho = 0.36, *p* < 0.001). There was also significant correlation of placebo effect-2 on mouth-dryness and drowsiness (rho = 0.64, *p* < 0.001) and on mouth-dryness and nausea (rho = 0.62, *p* < 0.001).

### Time course of drug and placebo effects

As shown in Fig. 3 (e and f), placebo effect-2 started earlier than drug effect and was rather flat, drug effect peaked around 2–3 h, and interaction model-estimated drug effect started to decline earlier than conventionally-estimated drug effect.

### Drug*placebo interaction effect

Mean difference between placebo effect-1 (placebo and interaction effects) and placebo effect-2 (placebo effect alone) was 35.1 mm*hr (5.6 to 64.6, *p* = 0.02) for drowsiness, 23.8 mm*hr (2.4 to 45.2, *p* = 0.03) for mouth-dryness, and 12.4 mm*hr (0.2 to 24.6, *p* = 0.047) for nausea. Further, drug + interaction effect was larger than interaction model-estimated drug effect with a mean difference of 42.6 mm*hr (1.3 to 84.0, *p* = 0.04) for drowsiness, 28.7 mm*hr (−1.5 to 58.9, *p* = 0.06) for mouth-dryness, and 14.1 mm*hr (−0.4 to 28.5, *p* = 0.06) for nausea. Furthermore, the total effect was larger than the sum of the interaction model-estimated drug effect and placebo effect-2 with a mean difference of 40.6 mm*hr (−2.3 to 83.5, *p* = 0.06) for drowsiness, 28.8 mm*hr (−3.7 to 61.4, *p* = 0.08) for mouth-dryness, and 12.8 mm*hr (−2.4 to 27.9, *p* = 0.09) for nausea.

The total effect on drowsiness, mouth-dryness, and nausea was 139.7 mm*hr (109.8 to 169.6), 63.6 mm*hr (41.1 to 86.1), and 20.8 mm*hr (9.4 to 32.1), respectively (Fig. 4 (d to f)). Therefore, it can be calculated that the drug*placebo interaction effect accounted for about 25, 37, and 60% of the total effect on drowsiness, mouth-dryness, and nausea.

## Discussion

We used a hybrid of balanced placebo and RPCT designs to determine the drug*placebo interaction effect (predefined primary objective), and 2) compare model-estimated drug effect obtained from the balanced placebo design to conventionally estimated drug effect obtained from the RPCT design (predefined secondary objective). We studied hydroxyzine pharmaceutical and placebo effects on healthy volunteers and measured drowsiness (therapeutic outcome), mouth-dryness (adverse outcome), nausea (positive control outcome for the placebo effect), and itchiness (negative control outcome for the placebo and drug effects). We found statistically significant and clinically important positive interaction effect on drowsiness, mouth-dryness, and nausea and that conventionally estimated drug effect is larger than model-estimated drug effect.

The specific placebo effect (meaning response) is often difficult to separate from the more general placebo response that may involve regression to the mean, natural course, Hawthorne effect, and the general effect of patient-physician relation (such as sympathy) [1–3, 16]. The placebo effect is not restricted to inert substances; it contributes importantly to total medication effect [7, 9, 17] and is lost with a false belief of receiving an inert substance [18].

Drug and placebo effects, just like any other effects, may interact. Since the medication arm, but not the placebo arm, of RPCT would have the interaction effect, the assumption that the difference between the two arms represents the drug effect [4, 16] may be incorrect. In this study, we found that the placebo effect was significantly larger when measured using hydroxyzine (described as hydroxyzine or placebo) than when measured using placebo (described as hydroxyzine or placebo). The first measurement includes both the placebo and interaction effects, whereas the second includes only the placebo effect. Further, the combination of placebo and drug effects was smaller than the total effect. Furthermore, conventionally estimated drug effect, which includes the drug effect plus part of the interaction effect, was larger than model-estimated drug effect. In a previous study, we showed that there may be negative drug*placebo interaction effect of caffeine on energy and sleepiness [9]. More recently, others showed significant negative drug*placebo interaction effect of lidocaine on experimental pain [7].

Both size and direction of interaction effects depend on whether causal contrasts are estimated on additive (difference) or multiplicative (ratio) scale. Although both the current and previous studies used the additive scale, the current study is the first to show positive interaction effect. Negative interaction may be related to ceiling of the effect, whereas, positive interaction may be related to sub-maximum drug and placebo effects or different underlying mechanisms.

The study makes two other interesting observations. We found drug*placebo interaction effect on mouth-dryness and nausea despite the absence of significant model-estimated drug effect, which suggests that interaction of effectors may not be restricted to be on the same outcome; drug effect on drowsiness appears to have interacted with the placebo effect on mouth-dryness and nausea (cross-outcome interaction). We also found that response to hydroxyzine given as unknown (with 50% possibility of being hydroxyzine) was intermediate between the response to open hydroxyzine and hidden hydroxyzine, suggesting that the relationship between the degree of subject’s certainty about receiving an intervention and the placebo effect is rather linear. This is in agreement with a recent review of indirect evidence [8] but in contrast to what was previously hypothesized [19].

### Limitations

Intervention’s administration by an undeceived investigator may have reduced the placebo effect and consequently the interaction effect. However, post-study debriefing revealed that 91% of participants believed what they were told at the time of intervention and 80% at all times. On the other hand, the balanced placebo design is prone to experimental subordination bias that might exaggerate the placebo response [16]. Since it was emphasized to participants that hydroxyzine effects do not occur all the time or in all persons, participants knew that coordinators were blinded to their assignments, and there was no patient-physician relationship or even further contact between the investigator who administered the intervention and the participants, such bias would be small. Further, biases related to the estimation of the placebo effect would not be expected to influence the primary conclusion of the study, whether or not an interaction effect exists. Another limitation of the study design is the learning effect, which is present in all crossover designs. Thus, deception and blinding would not be expected to be completely successful. However, the results of our post-study debriefing indicate that this limitation is rather minor and not likely to have a major effect on our conclusions. Finally, our study was performed on healthy volunteers using a small dose of hydroxyzine and it is not clear how much of the observed interaction effect would be present in RPCT on patients. Nevertheless, current interpretation of RPCT results is based on a model that assumes that there is no interaction between drug and placebo effects. Finding one example where such assumption is falsified is enough to question the model.

### Implications

The possibility that there may be drug*placebo interaction effect has important implications. For example, a negative interaction effect may explain the trivial antidepressants effect observed in most clinical trials [4, 9]. Alternatively, the observed trivial effect may be entirely due to cross-outcome interaction between the placebo effect on depression and side effects of antidepressants, with no antidepressant drug effect. Further, when comparing drug effects across RPCTs, it may be important to control for the interaction effect, since the placebo effect and hence the interaction effect may not be the same across trials. Furthermore, differences between the two arms of active-control trials may be related in part to differential interaction effect rather than purely to differential drug effect. Moreover, unblinding in RPCTs, would bias estimated drug effect not only because of differential placebo effect in the two arms but also because of different interaction effect. In addition, because of the potential cross-outcome interaction effect, active placebos may be more accurate than inert placebos in determining drug effects; partial placebo and partial cross-outcome interaction effects in the active placebo arm would cancel out the partial placebo and partial interaction effects in the drug arm. Finally, estimating the placebo effect by the open-hidden active drug design may be biased since the difference between the two arms would include an interaction effect.

## Conclusions

In this large hybrid design study, using hydroxyzine as a model drug, we found that there is a significant and important positive drug*placebo interaction effect on additive scale and that conventional measurement of drug effect size by randomized placebo-controlled trials may be biased.

## Abbreviations

AUC: Area-under-the-curve
KFSH & RC: King Faisal Specialist Hospital and Research Center
RPCT: Randomized placebo-controlled trial
VAS: Visual analog scale
